# Ovariotomy

**Published:** 1872-06

**Authors:** 


### Editorial.

### Ovariotomy,—Looking After cur Laurels.

It will be remembered that a few months since, we noticed the favor with which our idea of enucleating ovarian and other intra pelvic tumors, had been received in England, and the success there attained by this method. We have recently received the new edition of Thomas on the Diseases of Woman, and are much gratified by the compliment paid enucleation in ovariotomy by this distinguished teacher and author. He says: "I have resorted to this method advised by Dr. Miner of Buffalo, N. Y., three times, with good results, in cases which would have proved unmanageable by other means. It appears to me to to be one of the most valuable of all the contributions to ovariotomy, which have emanated from this country." This endorsement of our suggestion first made to the profession not more than three years since, and the universal favor with which experts in ovariotomy have considered the novel, and at first startling proposition that ovarian and other intra-pelvic tumors could be removed without hæmorrhage, and without cantery, clamp or ligature; thus leaving no pedicle to treat, and avoiding many of the difficulties and dangers of the proceedure, has very naturally been a source of pride and pleasure. If our readers do not have quite so much interest in it as we do, we will gladly excuse them; if in ovariotomy they will try the plan by enucleation.

